# Microbiota in Exhaled Breath Condensate and the Lung

**DOI:** 10.1128/AEM.00515-17

**Published:** 2017-05-31

**Authors:** Laura Glendinning, Steven Wright, Peter Tennant, Andrew C. Gill, David Collie, Gerry McLachlan

**Affiliations:** The Roslin Institute and Royal (Dick) School of Veterinary Studies, University of Edinburgh, Edinburgh, Midlothian, United Kingdom; University of Bayreuth

**Keywords:** 16S rRNA, EBC, colistimethate sodium, colistin, lung microbiota, respiratory microbiota, sheep

## Abstract

The lung microbiota is commonly sampled using relatively invasive bronchoscopic procedures. Exhaled breath condensate (EBC) collection potentially offers a less invasive alternative for lung microbiota sampling. We compared lung microbiota samples retrieved by protected specimen brushings (PSB) and exhaled breath condensate collection. We also sought to assess whether aerosolized antibiotic treatment would influence the lung microbiota and whether this change could be detected in EBC. EBC was collected from 6 conscious sheep and then from the same anesthetized sheep during mechanical ventilation. Following the latter EBC collection, PSB samples were collected from separate sites within each sheep lung. On the subsequent day, each sheep was then treated with nebulized colistimethate sodium. Two days after nebulization, EBC and PSB samples were again collected. Bacterial DNA was quantified using 16S rRNA gene quantitative PCR. The V2-V3 region of the 16S rRNA gene was amplified by PCR and sequenced using Illumina MiSeq. Quality control and operational taxonomic unit (OTU) clustering were performed with mothur. The EBC samples contained significantly less bacterial DNA than the PSB samples. The EBC samples from anesthetized animals clustered separately by their bacterial community compositions in comparison to the PSB samples, and 37 bacterial OTUs were identified as differentially abundant between the two sample types. Despite only low concentrations of colistin being detected in bronchoalveolar lavage fluid, PSB samples were found to differ by their bacterial compositions before and after colistimethate sodium treatment. Our findings indicate that microbiota in EBC samples and PSB samples are not equivalent.

**IMPORTANCE** Sampling of the lung microbiota usually necessitates performing bronchoscopic procedures that involve a hospital visit for human participants and the use of trained staff. The inconvenience and perceived discomfort of participating in this kind of research may deter healthy volunteers and may not be a safe option for patients with advanced lung disease. This study set out to evaluate a less invasive method for collecting lung microbiota samples by comparing samples taken via protected specimen brushings (PSB) to those taken via exhaled breath condensate (EBC) collection. We found that there was less bacterial DNA in EBC samples compared with that in PSB samples and that there were differences between the bacterial communities in the two sample types. We conclude that while EBC and PSB samples do not produce equivalent microbiota samples, the study of the EBC microbiota may still be of interest.

## INTRODUCTION

The study of the lung microbiota is a relatively new field in comparison to other areas of microbiota research. Although an increasing number of studies are linking changes in the composition of the lung bacterial communities to various disease states, including allergies, autoimmune disorders, and inflammatory and infectious diseases (1), the protocols for studying the lung microbiota are not standardized, making comparisons between studies difficult.

One issue with studying the lung microbiota is the invasiveness of the sampling techniques; the most common techniques are bronchoalveolar lavage (BAL) and the collection of protected specimen brushings (PSB), both of which require the subject to undergo bronchoscopy. The inconvenience and fear of complications associated with bronchoscopic procedures can result in healthy and/or diseased individuals declining to take part in studies (2), leading to a reduction in the potential pool of volunteers for lung microbiota studies. It is also currently unknown whether these sampling methods themselves can lead to changes in the lung microbiota.

Exhaled breath condensate (EBC) collection could potentially be a less invasive method for taking lung microbiota samples. This method involves condensing exhaled vapor into a liquid and has previously been used to study exhaled bacteria, viruses, and fungi (3–8). However, there have been no studies using 16S rRNA gene sequencing to compare the bacteria found in EBC samples to those found in samples taken directly from the lungs. Therefore, it is not known whether it can be used as a surrogate for more-invasive sampling techniques. We sought to assess the feasibility of using EBC in sheep to study the lung microbiota composition. We have previously used sheep as a model for studying the lung microbiota (9, 10) due to the anatomical and immunological similarity of their lungs to those of humans (11–13). In this study, we compared EBC samples collected from conscious sheep and from the same sheep under anesthesia to PSB samples taken from four spatially disparate sites within the lungs.

We then extended this to address whether EBC analysis has the capacity to detect changes in bacterial community compositions by attempting to directly manipulate the lung microbiota with an inhaled antibiotic (colistimethate sodium [CMS], which is active against Gram-negative bacteria). In a previous study, we examined the effect of intravenous CMS on the lung microbiota (9). While we did identify changes in the lung microbiota composition, the longer-term systemic antibiotic treatment used in that study also likely affected the gut microbial populations. Immunological links between gut and lung immunities, the gut-lung axis, raise the possibility that such changes may have indirectly influenced the lung microbiota (14). In this study, we delivered nebulized CMS, since this has been shown to lead to lower colistin plasma concentrations than injected CMS (15), enabling us to discern the direct effect of antibiotic treatment on respiratory bacterial communities.

A far greater quantity of bacterial DNA was isolated from PSB samples relative to EBC samples. We found that while there was some overlap between the types of bacteria found in these samples, EBC samples did cluster separately from PSB samples by their bacterial community compositions. Lastly, despite our antibiotic treatment regime only producing low concentrations of colistin in the lung epithelial lining fluids (the prodrug CMS is hydrolyzed *in vivo* to the active form of the drug, colistin), significant differences in community compositions were found between PSB samples derived pre- and posttreatment.

## RESULTS

### Analysis of sequence quality and controls.

DNA was extracted from respiratory samples and controls, and the V2-V3 variable regions of the 16S rRNA gene were amplified by PCR and then sequenced. After forming contigs from forward and reverse reads, various quality control steps were undertaken, which reduced the total sequence numbers by 25.8%. The lowest Good's coverage estimate value among the samples was 0.996, indicating that at least 99.6% of the bacteria in this sample were identified. The sequence error rate was 0.18% and the average number (± standard deviation [SD]) of reads per sample was 39,195 ± 11,535. In total, 867 operational taxonomic units (OTUs) were identified.

The Human Microbiome Project mock community HM-783D, containing the 16S rRNA genes of 20 bacterial species in staggered quantities and fixed ratios (1,000 to 1,000,000 copies per organism per μl), was processed alongside the samples. Some biases were identified (see Data Set S2 in the supplemental material). Three species were incorrectly identified at the species level (Acinetobacter baumannii was misidentified as Acinetobacter rhizosphaerae, Clostridium beijerinckii was misidentified as Clostridium butyricum, and Neisseria meningitidis was misidentified as Neisseria cinerea). Two of the bacterial species which were present in low numbers in the original community were not identified at any taxonomic level, namely, Actinomyces odontolyticus and Bacteroides vulgatus. Their absence is likely due to the fact that they were in low abundance rather than the inability of our protocol to amplify and identify them, as they have previously been identified using the same protocol on a nonstaggered version of the same mock community (10). We were also previously able to identify Enterococcus faecalis at the genus level, whereas in this study, it could not be identified except potentially as OTU 10, Bacilli (class). This discrepancy, combined with the fact that E. faecalis is in low abundance in the staggered mock community, leads us to believe that this identification is incorrect.

**FIG 1:**
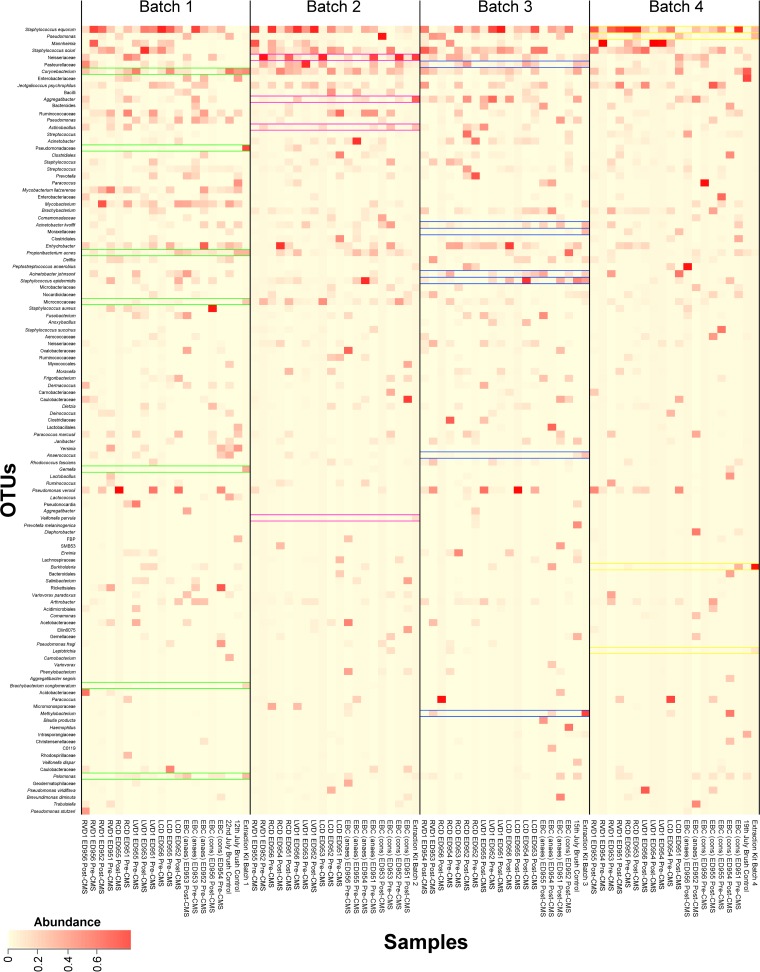
Heatmap showing samples grouped by batch based on the time DNA was extracted from the samples. Bacterial OTUs were included where they had an abundance of ≥5% in at least one sample. OTUs which were ≥5% abundant in a DNA extraction kit reagent control are indicated by color (batch 1, green; batch 2, pink; batch 3, blue; and batch 4, yellow). DNA extraction kit reagent controls are labeled as Extraction Kit Batch 1 to 4. EBC samples from conscious and from anesthetized sheep are labeled EBC (cons) and EBC (anaes), respectively. OTUs which were >5% abundant in an extraction kit control do not consistently appear in all samples in the same batch.

As lung bacteria are in low abundance, lung samples are at a particular risk for contamination by bacterial DNA originating from DNA extraction kit reagents. Therefore, as well as mock community controls, DNA extraction kit reagent controls were produced. DNA was extracted from samples in four batches and a reagent control was included with every batch. The bacterial OTUs identified in the extraction kit controls did not occur consistently in samples from the same batch (Fig. 1). Samples were clustered by DNA extraction batch (*P* < 0.001 by analysis of molecular variance [AMOVA]), and 30 OTUs were found to be indicative of specific batches (see Data Set S3). However, when these OTUs were removed from the data set, samples still clustered by extraction batch (*P* = 0.014 by AMOVA), indicating that clustering was not entirely due to the presence of these OTUs. It is possible that some of these OTUs may be found naturally within the sheep respiratory system (e.g., Micrococcus luteus, a common colonizer of the human upper respiratory tract). Therefore, we decided not to remove these OTUs from our data set. Since samples were randomly assigned to extraction batches, clustering by batch would be unlikely to lead to false-positive statistical results. However, there is the possibility that the presence of contaminating organisms may increase the heterogeneity and thereby also increase stochastic noise.

In controls, the most abundant OTUs on average were as follows: Corynebacterium, 14.4%; Enterobacteriaceae, 10.9%; and Intrasporangiaceae, 3.6% in PSB controls and Burkholderia, 14.0%; Neisseriaceae, 10.5%; and Aggregatibacter, 7.7% in DNA extraction kit reagent controls. The most abundant OTUs (on average) in the different sample types were as follows: Staphylococcus equorum, 10.7%; Mannheimia, 6.5%; and Staphylococcus sciuri, 5.6% in PSB samples; Staphylococcus equorum, 5.5%; Neisseriaceae, 4.7%; and Paracoccus, 4.3% in EBC samples from conscious sheep (cons); and Staphylococcus equorum, 5.1%; Staphylococcus epidermidis, 3.7%; and Peptostreptococcus anaerobius, 3.2% in EBC samples from anesthetized sheep (anaes).

### PSB samples contain more bacterial DNA than EBC samples.

**FIG 2:**
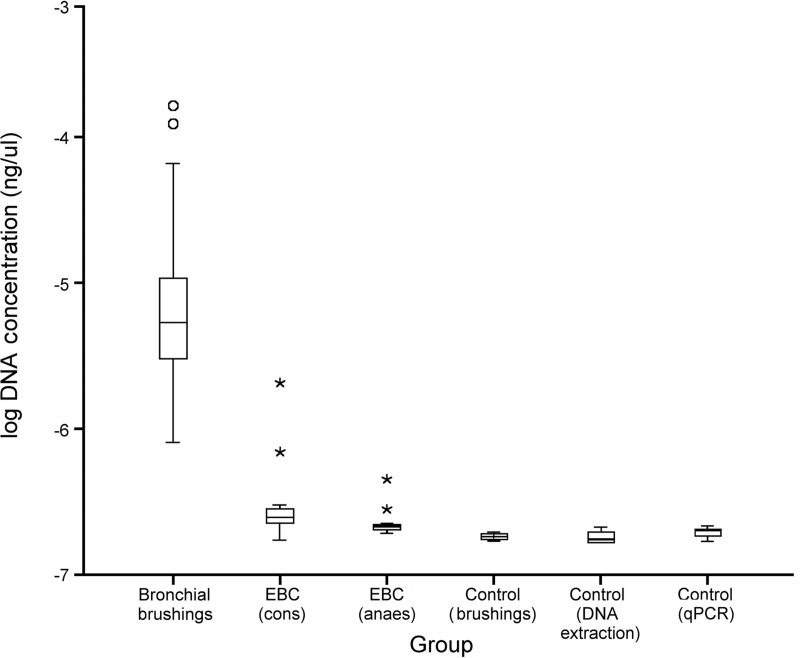
Boxplot showing the log 16S rRNA gene concentrations found in sheep respiratory samples (EBC samples from conscious and anesthetized animals and PSB samples) and controls (protected specimen brushes, DNA extraction kit reagents and qPCR reagents). Outliers were defined by SPSS as either “out” values (circles) or “extreme” values (stars). PSB samples contained significantly more bacterial DNA (*P* < 0.005) than any other respiratory sample type or control.

The V3 region of the 16S rRNA gene was quantified in our samples using quantitative PCR (qPCR). On average, PSB samples contained 1.53 × 10^−5^ ± 2.96 × 10^−5^ ng/μl (mean ± SD) bacterial DNA (34,200 ± 66,100 16S copy numbers/μl), while EBC samples from conscious and anesthetized sheep contained 4.28 × 10^−7^ ± 5.34 × 10^−7^ ng/μl (955 ± 1,190 16S copy numbers/μl) and 2.38 × 10^−7^ ± 7.12 × 10^−8^ ng/μl (531 ± 159 16S copy numbers/μl), respectively (Fig. 2). DNA extraction kit reagent controls contained 1.82 × 10^−7^ ± 2.21 × 10^−8^ ng/μl (406 ± 49 16S copy numbers/μl), while PSB controls and qPCR water controls contained 1.84 × 10^−7^ ± 1.05 × 10^−8^ ng/μl (411 ± 23 16S copy numbers/μl) and 1.98 × 10^−7^ ± 2.06 × 10^−8^ ng/μl (442 ± 46 16S copy numbers/μl), respectively.

All respiratory samples contained significantly more DNA than the controls (*P* < 0.005 for all sample types by Mann-Whitney U test). EBC samples from conscious and anesthetized animals did not contain significantly different quantities of DNA (*P* = 0.182 by Wilcoxon signed-rank test); however, PSB samples contained significantly more DNA than both EBC (cons) (*P* = 0.002 by Wilcoxon signed-rank test) and EBC (anaes) (*P* = 0.002 Wilcoxon signed-rank test) samples.

### No significant clustering of EBC by sampling method.

**FIG 3:**
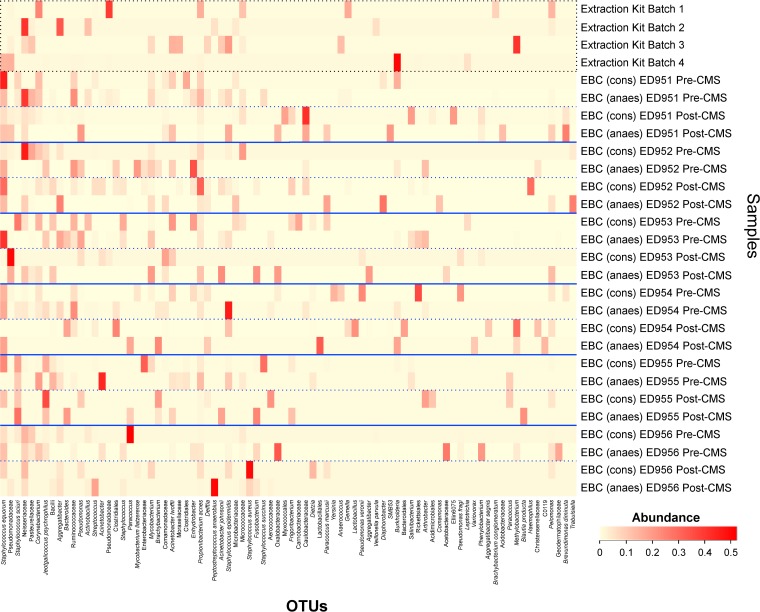
Heatmap showing EBC samples grouped by sheep and time point. DNA extraction kit reagent controls are labeled as Extraction Kit Batch 1 to 4. EBC samples from conscious and anesthetized sheep are labeled EBC (cons) and EBC (anaes), respectively. Bacterial OTUs were included where they had an abundance of ≥5% in at least one sample. As can be observed, EBC samples taken from the same sheep when it was conscious and when it was anesthetized did not necessarily contain the same bacterial OTUs.

Since EBC samples from conscious sheep would be expected to include more bacteria from the upper respiratory tract than EBC samples from anesthetized sheep, it was expected that these two groups of samples would cluster separately from one another. However, no significantly separate clustering was observed (*P* = 0.994 by AMOVA). Despite this lack of separate clustering, EBC samples taken from the same sheep while it was conscious or anesthetized did not contain the same bacterial communities, as can be observed in Fig. 3.

The richness and the diversity of bacterial communities were not significantly different between the two groups (*P* = 0.583 and *P* = 0.595, respectively, by Wilcoxon signed-rank test). When examined using Metastats, there were significant differences in the quantities of several OTUs between these groups, but all of these OTUs were present at low abundances (<1% abundant on average in each group).

### PSB samples and EBC (anaes) samples cluster separately by their bacterial communities.

**FIG 4:**
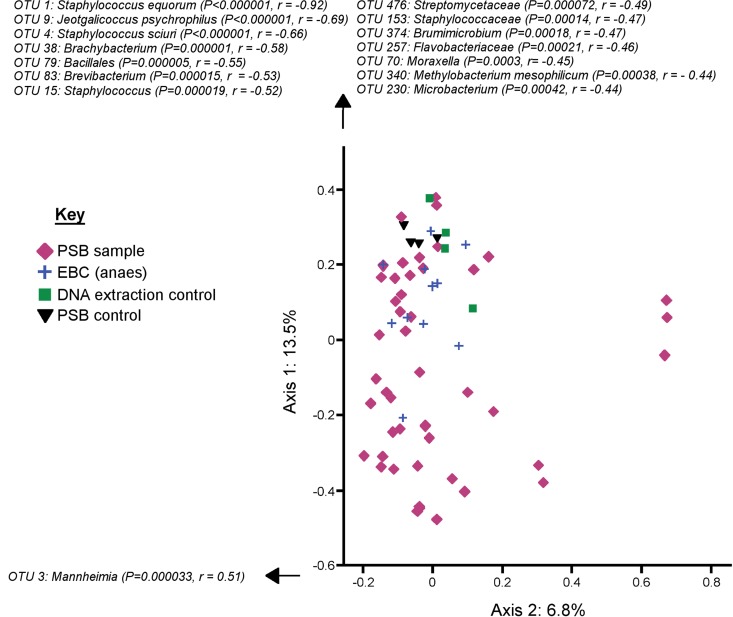
PCoA graph showing the significantly separate clustering of EBC (anaes) and PSB samples from sheep (*P* = 0.011 by AMOVA), which may be due to the difference in variation between the two sample types (*P* = 0.026 by HOMOVA). The OTUs which most contributed to samples moving in a positive or negative direction along either axis and which had *P* values of < 0.00058 (defined by Bonferroni's correction as 0.5 divided by the total number of OTUs), according to the corr.axes command within mothur, are listed. As this graph is only representative of 20.3% of the total variability present between samples, caution should be taken when interpreting how clustered the sample groups appear.

We next investigated whether PSB and EBC samples contained equivalent bacterial communities. We compared PSB samples with EBC (anaes) samples as we hypothesized that these would be less likely to be contaminated by upper respiratory tract microbes than EBC (cons) samples. As well as containing a larger quantity of bacterial DNA, PSB samples also contained bacterial communities that were significantly different from those of the EBC (anaes) samples (*P* = 0.011 by AMOVA) (Fig. 4). This may be explained by the difference in variation between the two groups (*P* = 0.026 by homogeneity of molecular variance [HOMOVA]). Bacterial communities from PSB samples were also found to be significantly richer (*P* = 0.006 by Wilcoxon signed-rank test), but there was no significant difference in diversity (*P* = 0.48 by Wilcoxon signed-rank test). One OTU designated Pseudomonas veronii, which was the 4th most abundant OTU in PSB samples, was found to be significantly more abundant in PSB samples (PSB samples [mean ± SD], 3.9% ± 1.3%; EBC, only one sequence read found in one sample; Metastats *q* value = 0.046). The P. veronii OTU was not found in any of the PSB controls, indicating that its presence is not likely due to contamination. This indicates that the EBC samples do not simply contain a subset of the most abundant OTUs from PSB samples. An additional 36 low-abundance OTUs (<1% abundant on average in either group) were found to be significantly different between the two groups by Metastats.

We considered that since EBC (anaes) samples contained far less bacterial DNA than PSB samples, they may have been more affected by contamination and this may be why these sample types clustered separately. However, the five most abundant OTUs found in DNA extraction kit reagent controls (Burkholderia, Neisseriaceae, Aggregatibacter, Pseudomonadaceae, and Methylobacterium) were not found to be significantly differently represented between PSB samples and EBC (anaes) samples (Metastats *q* value = 1). Therefore, it seems unlikely that the separate clustering of these groups was due merely to the increased effect of contamination on EBC (anaes) samples.

### Changes in the bacterial communities found in respiratory samples before and after CMS treatment.

**FIG 5:**
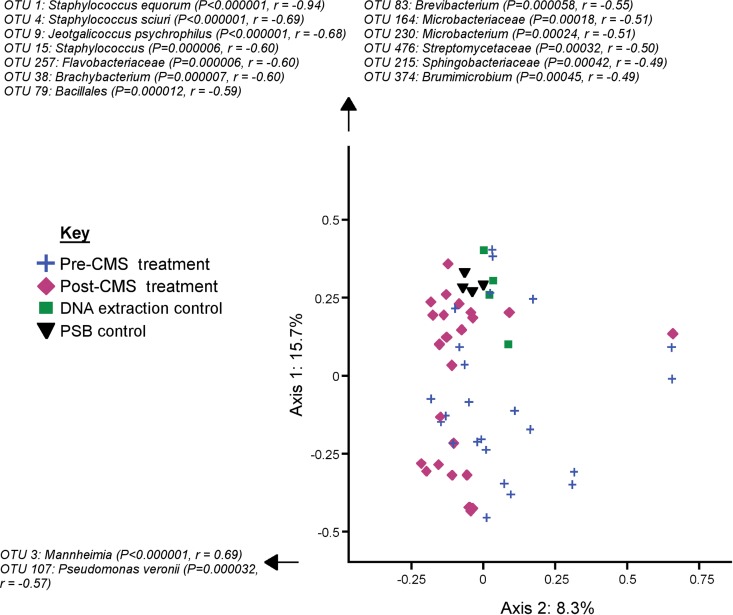
PCoA graph showing the significantly separate clustering of PSB samples from sheep before and after CMS treatment (*P* = 0.014 by AMOVA). The OTUs which most contributed to samples moving in a positive or negative direction along either axis and which had *P* values of < 0.00058 (defined by Bonferroni's correction as 0.5 divided by the total number of OTUs), according to the corr.axes command within mothur, are listed. As this graph is only representative of 24% of the total variability present between samples, caution should be taken when interpreting how clustered the sample groups appear.

For both EBC (cons) and EBC (anaes) samples, pre- and posttreatment samples did not differ significantly by bacterial community structure (*P* = 0.449 and *P* = 0.094, respectively, by AMOVA). However, the bacterial communities found in PSB samples were found to be significantly different pre- and posttreatment (*P* = 0.014 by AMOVA) (Fig. 5). This significantly separate clustering was not merely due to differences in variation between the two groups (*P* = 0.87 by HOMOVA). The OTU P. veronii was increased in posttreatment samples (pretreatment [mean ± SD], 0.74% ± 0.39%; posttreatment, 7.1% ± 2.4%; Metastats *q* value = 0.043), and a further 97 low-abundance (<0.1%) OTUs were found to significantly differentiate pre- and posttreatment samples.

Using the Wilcoxon signed-rank test, it was found that the concentrations of DNA in respiratory samples before and after CMS treatment did not differ significantly: PSB samples, *P* = 0.689; EBC (cons) samples, *P* = 0.345; and EBC (anaes) samples, *P* = 0.248. The concentrations of colistin A identified in sheep lungs are shown in Table 1.

**TABLE 1:** Colistin A concentrations in sheep epithelial lining fluid

Sheep	Colistin A concn^*a*^ (ng/μl)	Dilution of epithelial lining fluid in BAL	Mean colistin A concn corrected for dilution (ng/μl)
ED951	0.346 ± 0.056	5.45	1.89
ED952	0.320 ± 0.034	4.18	1.34
ED953	0.290 ± 0.061	6.45	1.87
ED954	1.549 ± 0.251	15.75	24.40
ED955	0.625 ± 0.159	11.43	7.15
ED956	0.222 ± 0.017	29.5	6.56

a Values are the means ± SD. Colistin B values were too low to be calculated accurately.

## DISCUSSION

In this study, we sought to identify whether invasive lung microbiota sampling techniques could be replaced by a less invasive method. We compared the quantities of bacterial DNA and the bacterial communities from samples taken by PSB and EBC collection in six sheep at two sampling points. EBC was collected from both conscious and anesthetized animals. During mechanical ventilation, the animals were intubated, meaning that the exhaled breath collected was derived only from the lower respiratory tract. By comparing these samples to those taken from conscious animals, it should be possible to analyze the extent of contamination by bacteria from the upper respiratory tract in EBC (cons) samples. We found that EBC samples contained significantly less bacterial DNA than the PSB samples and that PSB samples clustered separately from EBC (anaes) samples by the composition of their bacterial communities. EBC (anaes) and EBC (cons) samples did not cluster separately from one another.

Studies examining the utility of EBC for identifying lung-colonizing microorganisms have shown variable results. A study comparing EBC and sputum samples from asthma patients showed a 100% overlap in the culturable fungi identified between the two sample types (5), and a study examining the bacterial pathogens cultured from BAL and EBC samples in patients with ventilator-associated pneumonia showed a high concordance between the two sampling methods (16). In comparison, when PCR assays for 10 common respiratory pathogens were performed on EBC and sputum samples from chronic obstructive pulmonary disease patients, the results were found not to correlate well (17). EBC collection has also previously been found to be inefficient for detecting Mycobacterium tuberculosis (18), influenza viruses (19), and the common cystic fibrosis pathogens Pseudomonas aeruginosa and Burkholderia cepacia (20).

Some concerns have been raised about the use of EBC in respiratory research, since the epithelial lining fluid contained in these samples is often variable and is very highly diluted with water vapor (21). This dilution could explain the far lower concentrations of bacterial DNA we identified in EBC samples in comparison to those from PSB samples. It is also likely that PSB would be more efficient for sampling biofilms adhered to the lung mucosa, which could explain some of the differences observed between the two sample types. The difference between the bacterial communities found in PSB and EBC samples may also be partially explained by how EBC is formed. The exact origin of EBC is still under debate, but it has been suggested that differences observed between BAL and EBC samples could be explained by the fact that different compartments of the lung are sampled (22). While it might be assumed that EBC would be derived from both the central and peripheral airway compartments, which would perhaps explain the differences between these samples and PSB samples, Bondesson et al. concluded that the majority of EBC is in fact derived from the central airways (23). Without a better understanding of how EBC is formed and what influences its composition, we are unable to account for the differences we observed between the two sampling types.

Despite the fact that the concentrations of colistin found in the lungs were quite low after nebulized CMS treatment, a significant difference was observed in the bacterial communities from PSB samples pre- and posttreatment. In a previous study, we found that the relative proportion of Gram-negative bacteria in the lung microbiota (excluding Pseudomonadales) was reduced after injected CMS treatment (9). However, members of Pseudomonadales generally increased in relative abundance or remained stable after treatment. Therefore, it is interesting to note that while in this study we did not find a significant reduction in the abundance of Gram-negative bacteria in PSB samples (data not shown), an OTU belonging to Pseudomonadales (P. veronii) was significantly increased in these samples after CMS treatment. It is possible that, even at low concentrations, the colistin may have had some effect on the lung bacteria or that the sampling strategy may itself in some way lead to changes in the lung microbiota, but at the moment, this is purely speculative. All samples were randomized prior to DNA extraction and PCR amplification; therefore, the observed differences were not due to samples from one time point being processed separately from those from the other time point.

In conclusion, the differences we observed between PSB samples and EBC samples lead us to not recommend using EBC collection as a replacement for more-invasive lung sampling techniques. However, the EBC microbiota may still be an interesting avenue of study despite the fact that the small quantities of bacterial DNA in these samples leave them more vulnerable to contamination, and any future studies would have to be designed with this in mind.

## MATERIALS AND METHODS

### Animals.

Six commercially sourced, castrated male Suffolk-cross sheep aged 14 months were used in this study. All animal experiments were approved by the Roslin Institute Animal Welfare and Ethics Committee and were subject to the Animals (Scientific Procedures) Act of 1986. Sheep had previously been housed outdoors as part of a large flock but were moved indoors before the study and remained indoors until the study end. Sheep were separated into two pens sharing the same airspace. One pen contained sheep ED951, ED952, and ED953, while the other contained sheep ED954, ED955, and ED956. The rectal temperatures and weights of all animals were taken prior to the initial respiratory tract sampling. The animals weighed on average (± SD) 49.2 ± 3.4 kg and the rectal temperatures were measured as 38.9 ± 0.89°C.

### Experimental design.

**FIG 6:**
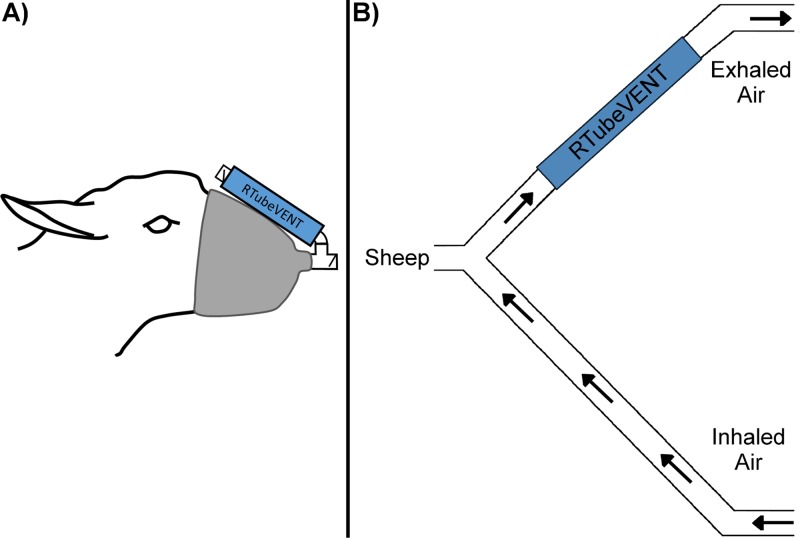
(A) EBC was collected from conscious animals while they were restrained in a yoke head-restraint holding crate. A face mask was attached and sheep inhaled through a short tube with an inlet valve and exhaled through an RTubeVENT. (B) EBC was collected from anesthetized mechanically ventilated animals by placing the RTubeVENT in-line with the expiratory limb of the ventilator, near the sheep's head.

Conscious animals were confined in a yoke head-restraint holding crate, and EBC was collected for 10 min using an RTubeVENT with a cooling sleeve (Respiratory Research, Charlottesville, VA, USA) attached to a face mask. The sheep inhaled through a one-way inspiratory valve and expired through the RTubeVENT (Fig. 6). The exhaled breath condensate samples from conscious sheep (EBC [cons]) were transferred from the RTubeVENT into Eppendorf tubes according to the manufacturer's instructions and were frozen on dry ice within an hour of collection.

**FIG 7:**
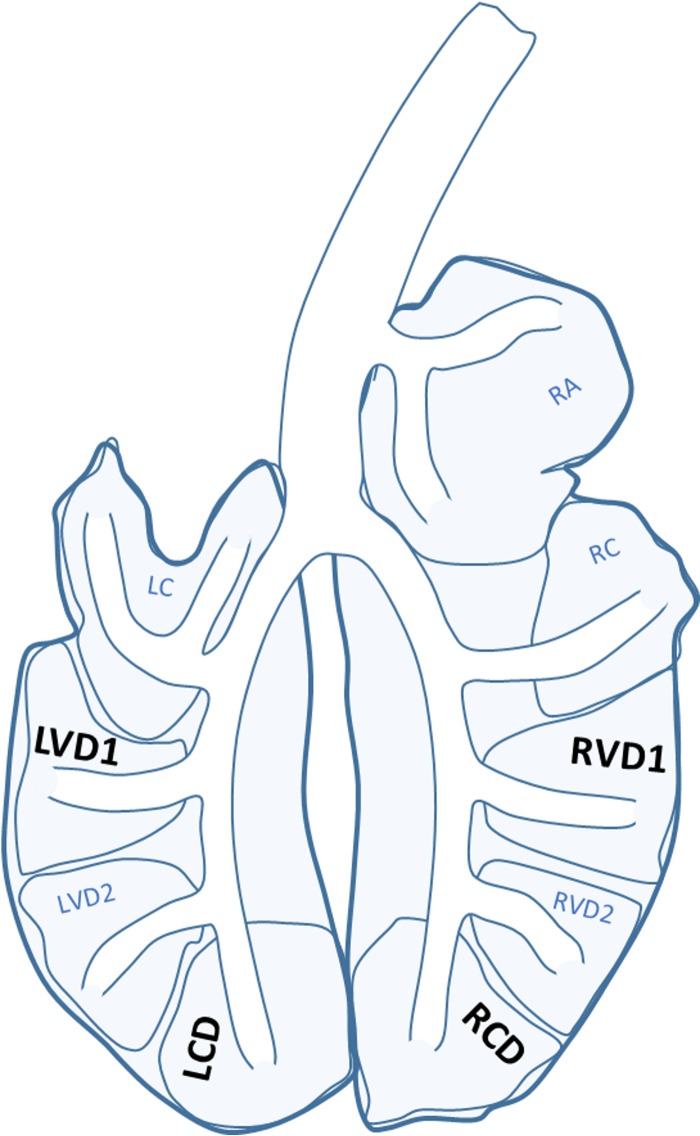
Diagram of the sheep lung. PSB samples were taken before and after colistimethate sodium treatment from the right ventral diaphragmatic 1 (RVD1), left ventral diaphragmatic 1 (LVD1), right caudal diaphragmatic (RCD), and left caudal diaphragmatic (LCD) lung segments. RC, right cardiac; RA, right apical; LC, left cardiac; LVD2, left ventral diaphragmatic 2; RVD2, right ventral diaphragmatic 2. Adapted from reference 24.

The sheep were then anesthetized (3 to 5 h later) according to a procedure that has previously been described (24). Bronchoscopy was performed using an endotracheal tube. During anesthesia, EBC samples were collected for 10 min by incorporating an RTubeVENT within the expiratory limb of the anesthetic circuit (Fig. 6). The condensate was again transferred into Eppendorf tubes. The exhaled breath condensate samples from the anesthetized sheep (EBC [anaes]) were frozen on dry ice within an hour of collection. PSB samples (disposable microbiology brush; Conmed, Utica, NY, USA) were taken from the left ventral diaphragmatic 1 (LVD1), right ventral diaphragmatic 1 (RVD1), right caudal diaphragmatic (RCD), and left caudal diaphragmatic (LCD) lung segments (Fig. 7). Brushes were cut into phosphate-buffered saline (PBS; Sigma-Aldrich, Irvine, UK) for storage. For each sampling day, an unused protected specimen brush was cut into PBS to act as a control.

Eighteen hours after the recovery from anesthesia, sheep were administered 2,000,000 IU of CMS in 4 ml distilled water by inhalation (Colomycin for injection; Forest Laboratories UK Ltd., Dartford, UK). Restraint of the conscious sheep was as described above, and the CMS was delivered using a face mask connected via the inspiratory limb to an eFlow rapid nebulizer (PARI Respiratory Equipment Inc., Midlothian, VA, USA). This treatment was repeated 6 h later. Two days after the first CMS dose was administered, EBC (cons), EBC (anaes), and PSB samples were again collected as described above. Sheep were killed by barbiturate overdose and exsanguination and blood samples were collected. Blood was centrifuged at 2,500 × *g* for 5 min and the serum was removed and frozen on dry ice. Immediately postmortem, 20-ml aliquots of PBS were used to collect BAL fluid. The urea concentrations in plasma and BAL fluid were used to calculate the dilution factor of lung epithelial lining fluid in BAL fluid (25).

### Quantitation of colistin in BAL fluid of sheep.

BAL fluid was centrifuged at 1,400 × *g* for 5 min to remove cells prior to colistin quantification. The quantitation of colistin in ovine BAL fluid essentially follows the method previously published by Marchand et al. (26). Briefly, colistin sulfate (item no. 17584 [mixture of A and B isoforms]; Cayman Chemicals, Ann Arbor, Ml, USA) was dissolved in H_2_O to 1 mg/ml and a series of 7 calibrant solutions were created by diluting the stock solution into blank BAL fluid to cover the range from 100 to 0.07 μg/ml. Polymyxin B (Sigma-Aldrich, Irvine, UK) was used as an internal standard and was dissolved in water to 300 μg/ml. Two microliters of internal standard was added to 200 μl of each of the calibrant solutions and to 200 μl of each of the test samples. Eight hundred microliters of a solution of H_2_O and 0.1% (vol/vol) formic acid was added to each of the samples/calibrants, and each was partially purified by binding to a DSC-18 SPE cartridge (Sigma-Aldrich, Irvine, UK) and eluted with 400 μl methanol (MeOH) and 0.1% (vol/vol) formic acid. The eluted fractions were dried under vacuum and reconstituted in 50 μl of H_2_O and 0.1% (vol/vol) formic acid for subsequent analysis.

All calibrants and samples were centrifuged at 13,000 × *g* for 5 min to pellet any precipitate and then were analyzed by online liquid chromatography tandem-mass spectrometry (LC-MS/MS) in duplicate. Aliquots of 5 μl were injected into an Ace Ultracore 2.5 SuperC18 high-performance liquid chromatography (HPLC) column (75 mm by 2.1 mm) preequilibrated with 98% (vol/vol) buffer A, where HPLC buffer A was H_2_O with 0.1% (vol/vol) formic acid and 0.01% (vol/vol) trifluoroacetic acid, while HPLC buffer B was acetonitrile with 0.1% (vol/vol) formic acid and 0.01% (vol/vol) trifluoroacetic acid. The HPLC separation was developed by the following steps: from 2% buffer B at 0 min to 18% buffer B at 1 min, 22% buffer B at 3.5 min, 100% buffer B at 4 min, 100% buffer B at 5 min, and returning to 2% buffer B at 6 min for 5 min to reequilibrate. The flow rate was 200 μl/min and the eluent was passed directly to the electrospray source of an Amazon ETD ion trap mass spectrometer (Bruker, Billerica, MA, USA) operated in positive-ion mode. The mass spectrometer was operated under multiple reaction monitoring conditions, using parent ions of 578.3, 585.3, and 602.3 (representing the double-charged ion of colistin B, colistin A, and polymyxin B, respectively), fragmentation amplitudes of 0.8, and cutoffs of 140 in each case. Calibration curves and colistin concentrations were calculated by Bruker's proprietary software QuantAnalysis using the following reporter ions: 526.3, 535.3, 567.3, and 576.3 (colistin A); 519.3, 528.3, 560.800, and 569.3 (colistin B); and 543.300, 552.300, 584.300, and 593.3 (polymyxin B).

### DNA extraction.

DNA extraction was carried out as described previously (10) using the Mo Bio PowerSoil DNA isolation kit (Mo Bio Laboratories Inc., Carlsbad, CA, USA). All DNA extractions were carried out using extraction kits from the same lot, as the contamination present in different lots of the same make of kit has been shown not to be consistent (27). Samples were randomly assigned to one of four DNA extraction batches, and for each of these batches, an extraction kit reagent-only control was produced (sample groupings can be found in Data Set S1 in the supplemental material).

### 16S rRNA gene amplification and sequencing.

The V2-V3 variable regions of the 16S gene were amplified as described previously (10). A nested PCR protocol was used to decrease the potential bias introduced by the use of barcoded primers by only including primers with Illumina adaptor sequences and barcodes in the second PCR round (28). The first round used the V1-V4 primers 28F (5′-GAGTTTGATCNTGGCTCAG-3′) and 805R (5′-GACTACCAGGGTATCTAATC-3′) and the second round used the V2-V3 primers 104F (5′-GGCGVACGGGTGAGTAA-3′) and 519R (5′-GTNTTACNGCGGCKGCTG-3′) with Illumina adaptor sequences and barcodes (Data Set S1). The PCR conditions for the first round were 94°C for 2 min followed by 20 cycles of 94°C for 1 min, 55°C for 45 s and 72°C for 1.5 min, followed by 72°C for 20 min. The conditions for the second round were 98°C for 30 s followed by 20 cycles of 98°C for 10 s, 67°C for 30 s, and 72°C for 10 s, followed by 72°C for 2 min. Q5 High-fidelity 2× master mix (New England BioLabs, Ipswich, MA, USA) was used for all reactions. After each PCR round, amplicons were purified using the AMPure XP PCR purification system (Beckman Coulter, Brea, CA, USA). The Human Microbiome Project mock community HM-783D (obtained through BEI Resources, NIAID, NIH) also underwent PCR alongside samples and controls. The Qubit dsDNA HS assay kit (Thermo Fisher Scientific, Hemel Hempstead, UK) was used to calculate the quantity of DNA in each sample, and then samples were pooled into a sequencing library. Sequencing was performed using Illumina MiSeq (Illumina, San Diego, CA, USA) producing 250-bp paired-end reads.

### Bioinformatic and statistical analysis.

Primers were removed with cutadapt (29) and sequences with greater than one base error per 10 bases were discarded. Quality control, taxonomic assignment, and OTU clustering were performed in mothur (30) as described previously (10). The data were subsampled to the minimum number of sequence reads found in one of our samples (11,675). Except where stated, the following analyses were all performed within mothur.

Good's coverage values were calculated to estimate sample coverage (31). Distance matrices were constructed using Yue-Clayton theta values (32), and AMOVA was used to compare groups of samples by their bacterial composition (33). HOMOVA was used to compare groups by their variation (34). Principal-coordinate analysis (PCoA) graphs were constructed to visualize sample clustering. The mothur command corr.axes was used to correlate bacterial OTUs to the axes of the PCoA graphs using the Spearman's rank correlation coefficient (*r*). Bonferroni's correction was used to correct for multiple statistical tests. The inverse Simpson's index was employed to measure microbial diversity and the Chao 1 index was employed to measure richness. Metastats was used to identify OTUs which were significantly different between groups (35) except where more than two groups were compared, in which case indicator analysis was used (36).

To compare groups statistically when data were nonparametric, the Mann-Whitney U test was used if the groups were independent and the Wilcoxon signed-rank test was used when samples were related (performed in SPSS Statistics 21; IBM Analytics). Boxplots for qPCR data were constructed in SPSS. Heatmaps were constructed in R (version 3.2.2; R Foundation for Statistical Computing) using the packages gplots (37), heatplus (38), RColorBrewer (39), and Vegan (40).

### qPCR.

Quantification of the V3 region of the 16S rRNA gene was carried out using a previously described method (10). A standard curve was generated using DNA extracted from Pseudomonas aeruginosa strain PA0579 using 9 serial dilutions ranging from 14.2 ng/μl to 1.42 × 10^−7^ ng/μl (quantified by the Qubit dsDNA HS assay). The 0.142 ng/μl dilution served as a positive control for all qPCRs. The average threshold cycle (*C_T_*) value of no-template controls was 28.7.

qPCR was performed using 1 μl of extracted DNA solution, the primers UniF340 (5′-ACTCCTACGGGAGGCAGCAGT-3′) and UniR514 (5′-ATTACCGCGGCTGCTGGC-3′) at a final concentration of 0.4 μM, and the LightCycler 480 SYBR green I master mix (Roche Applied Science, Mannheim, Germany). The qPCR run consisted of a preincubation step (50°C for 2 min and 95°C for 10 s), an amplification step (45 cycles of 95°C for 30 s and then 63°C for 30 s), and a melting cycle.

### Accession number(s).

Sequencing reads can be accessed under BioProject accession number PRJNA337937.

## Supplementary Material

Supplemental material
