# Supplementary material for: Microbiota in Exhaled Breath Condensate and the Lung
Source: Appl Environ Microbiol. 2017 May 31;83(12):e00515-17. doi: 10.1128/AEM.00515-17 (PMC5452816; doi:10.1128/AEM.00515-17)
Supplement: Supplemental material [file supp_83_12_e00515-17__index.html]

Supplemental material 

# Microbiota in Exhaled Breath Condensate and the Lung

## Supplemental material

- Supplemental file 1 -

  Sample details, including collection dates, DNA extraction batches, and primer sequences (Data Set S1).

  XLSX, 20K
- Supplemental file 2 -

  Expected versus actual sequence abundances in mock community (Data Set S2).

  XLSX, 13K
- Supplemental file 3 -

  OTUs indicative of samples from specific DNA extraction batches (Data Set S3).

  XLSX, 13K
